# Formation of 3D Human Osteoblast Spheroids Incorporating Extracellular Matrix-Mimetic Phage Peptides as a Surrogate Bone Tissue Model

**DOI:** 10.3390/ijms26178482

**Published:** 2025-09-01

**Authors:** Maria Giovanna Rizzo, Dario Morganti, Antonella Smeriglio, Emanuele Luigi Sciuto, Massimo Orazio Spata, Domenico Trombetta, Barbara Fazio, Salvatore Pietro Paolo Guglielmino, Sabrina Conoci

**Affiliations:** 1 Department of Chemical, Biological, Pharmaceutical and Environmental Sciences, University of Messina, Viale Ferdinando Stagno d’Alcontres, 31, 98166 Messina, Italy; antonella.smeriglio@unime.it (A.S.); emanueleluigi.sciuto@unime.it (E.L.S.); domenico.trombetta@unime.it (D.T.); sguglielm@unime.it (S.P.P.G.); 2 CNR-IMM, Institute for Microelectronics and Microsystems, Viale F. Stagno D’Alcontres 31, 98166 Messina, Italy; dario.morganti@unime.it (D.M.); barbara.fazio@cnr.it (B.F.); 3 Department of Mathematics and Computer, University of Catania, Viale A. Doria 6, 95125 Catania, Italy; massimo.spata@unict.it; 4 Department of Chemistry “Giacomo Ciamician”, University of Bologna, Via Selmi, 2, 40126 Bologna, Italy

**Keywords:** bone physiology, 3D bone tissue model, cell–cell interaction, cell communication, extracellular matrix, regenerative medicine

## Abstract

Cell–cell communication and extracellular matrix (ECM) organization in a bone microenvironment are essential to replicate the bone microenvironment accurately. In this study, the extracellular matrix (ECM) was emulated by incorporating M13 phages, selected through phage display for displaying engineered peptides that mimic bone matrix proteins, into human osteoblast cultures to develop a three-dimensional bone model (3D BMP-Phage). Comprehensive analysis was performed to investigate: (i) the morphological development of spheroids, assessed by optical microscopy and quantified via fractal dimension analysis using box-counting algorithms; (ii) the biochemical composition of the extracellular matrix, evaluated by Raman spectroscopy; (iii) ECM protein deposition, analyzed through immunofluorescence staining; (iv) matrix mineralization, assessed by Alizarin Red staining and alkaline phosphatase (ALP) activity assay; and (v) osteogenic gene expression, measured by quantitative RT-PCR. The findings demonstrate that the 3D BMP-Phage model, facilitated by a cocktail of bone-mimicking peptides, enhances structural integrity, ECM complexity, mineralization, and osteogenic pathways compared to the control. This novel approach replicates key aspects of the bone microenvironment, providing a valuable platform for advanced physiological and regenerative medicine research under controlled conditions.

## 1. Introduction

Bone physiology research and regenerative medicine share the common goal of understanding and restoring the structural and functional integrity of skeletal tissues [1,2]. In this context, the application of engineered peptides in regenerative medicine has gained increasing attention due to their high biocompatibility, tunability, and ability to mimic functional motifs of native extracellular matrix (ECM) proteins [3]. Peptides offer key advantages over conventional macromolecules, including reduced immunogenicity, ease of chemical synthesis, and the potential for targeted interactions with cell surface receptors. In tissue engineering, bioactive peptides have been employed to enhance cell adhesion, migration, and lineage-specific differentiation, making them versatile tools for promoting the regeneration of complex tissues [4,5].

From a physiological perspective, such peptides can reproduce essential biochemical cues of the native environment, activating intracellular pathways involved in osteoblast proliferation, differentiation, and matrix mineralization [6].

Among musculoskeletal tissues, bone regeneration presents specific challenges due to the requirement for coordinated cellular interactions, ECM organization, and mineralization. Bone is a hierarchically structured tissue composed of osteoblasts, osteoclasts, and osteocytes embedded within a dynamic ECM rich in collagen, glycoproteins, and signaling molecules. The ECM provides not only mechanical support but also regulatory cues that influence cell fate, proliferation, and differentiation. Its continuous remodeling, mediated by the balanced activity of osteoblasts and osteoclasts, ensures skeletal integrity and mineral homeostasis throughout life [7]. Thus, replicating this intricate environment in vitro is crucial to model bone physiology and pathology accurately [4,8,9,10].

The development of three-dimensional (3D) models, including spheroids and organoids, is revolutionizing biomedical research by offering more reliable alternatives to traditional two-dimensional (2D) cultures [11]. Unlike 2D systems, 3D cultures allow cells to grow and interact within their environment in three dimensions, closely mirroring in vivo tissue conditions. In 3D models, cell–cell and cell–ECM interactions are enhanced, ensuring cell proliferation, differentiation, and morphologies that better resemble physiological conditions [12]. To mimic the functional complexity of the native ECM, recent advances have demonstrated that bioactive peptides derived from bone-specific proteins and growth factors can promote osteogenic differentiation and support bone tissue formation [13]. Such peptides often correspond to short amino acid motifs, known as mimotopes, capable of binding integrins, growth factor receptors, or other membrane proteins involved in osteogenic signaling cascades [14,15].

These peptides can promote osteogenic differentiation and support bone tissue formation when incorporated into biomaterials. Among the most versatile tools to identify and display such peptides is phage display (PD) technology, which allows the presentation of functional sequences on bacteriophage coat proteins [16,17]. Peptides selected through PD have shown high affinity for cell surface receptors, promoting cell adhesion, proliferation, and differentiation in osteochondral systems [18]. Phage display enables iterative selection (biopanning) under increasingly stringent conditions, ensuring the recovery of sequences with high specificity and functional relevance to the target cells. PD-derived peptides represent a promising strategy to engineer ECM-mimicking environments in 3D cell culture models [4,14]

This study aimed to develop a 3D in vitro model of human osteoblast spheroids incorporating a cocktail of phage-displayed peptides (BMP-Phage) selected for their sequence similarity with bone matrix proteins. The goal was to recreate a bone-like microenvironment that enhances cell aggregation, ECM deposition, and osteogenic differentiation. The model was characterized through morphological, spectroscopic, biochemical, and gene expression analyses to assess its structural and functional fidelity as a surrogate bone system.

## 2. Results

To generate a cocktail of bone-mimetic peptides, a PD biopanning strategy [19,20] was employed using primary human bone marrow-derived cells as the target. After multiple rounds of affinity selection, phage clones displaying peptide inserts on the major coat protein pVIII were isolated and individually propagated. The DNA inserts of the selected clones were sequenced, and the corresponding amino acid sequences were subjected to bioinformatic alignment to identify homologies with known bone extracellular matrix proteins. The analysis revealed that several peptide sequences shared mimetic similarity with key structural and regulatory proteins of the bone matrix, including Bone Morphogenetic Protein 2 (BMP-2), Osteonectin (SPARC), Integrin-Binding Sialoprotein (IBSP), Collagen alpha-1(I) chain, and Fibronectin. These assignments are summarized in Table 1.

**Figure 1:**
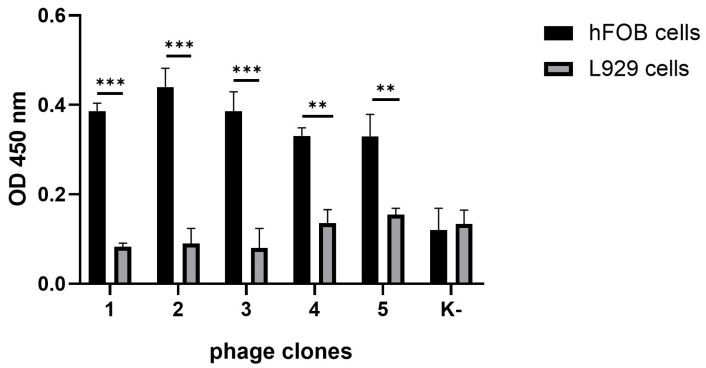
Binding specificity of selected phage clones. Absorbance at 450 nm (mean ± SD) following ELISA analysis of peptides-phage attachment to hFOB 1.19 vs. L929 cells. Phage clones with peptides *n*. 1–5 represent selected sequences with specificity for the osteoblastic lineage, obtained through biopanning. K– indicates a phage clone with non-specific sequence. Statistical analysis was performed using one-way ANOVA followed by Bonferroni post hoc test (** *p*-values < 0.01 *** *p*-values < 0.001). The data were derived from three independent experiments conducted in triplicate.

To evaluate the ability of the selected phages to interact specifically with bone-derived cells, a cellular ELISA was performed. Human fetal osteoblasts (hFOB 1.19) were used as the target population, while murine fibroblasts (L929) served as the negative control. As shown in Figure 1, all BMP-Phage clones exhibited significantly higher (*p*-values < 0.001) binding to hFOB cells compared to L929 cells, confirming their selective affinity for the osteoblastic lineage. Furthermore, a phage clone with non-specific sequence (K–) did not show appreciable binding to either cell type, further validating the effectiveness of biopanning in selecting phage-exposed peptides with high target specificity. These characterized phage clones were subsequently pooled into a composite BMP-Phage cocktail, which was employed to develop the 3D osteoblast spheroid model described in the following sections.

### 2.1. Three-Dimensional Human Osteoblast Model and Morphological Characterization

**Figure 2:**
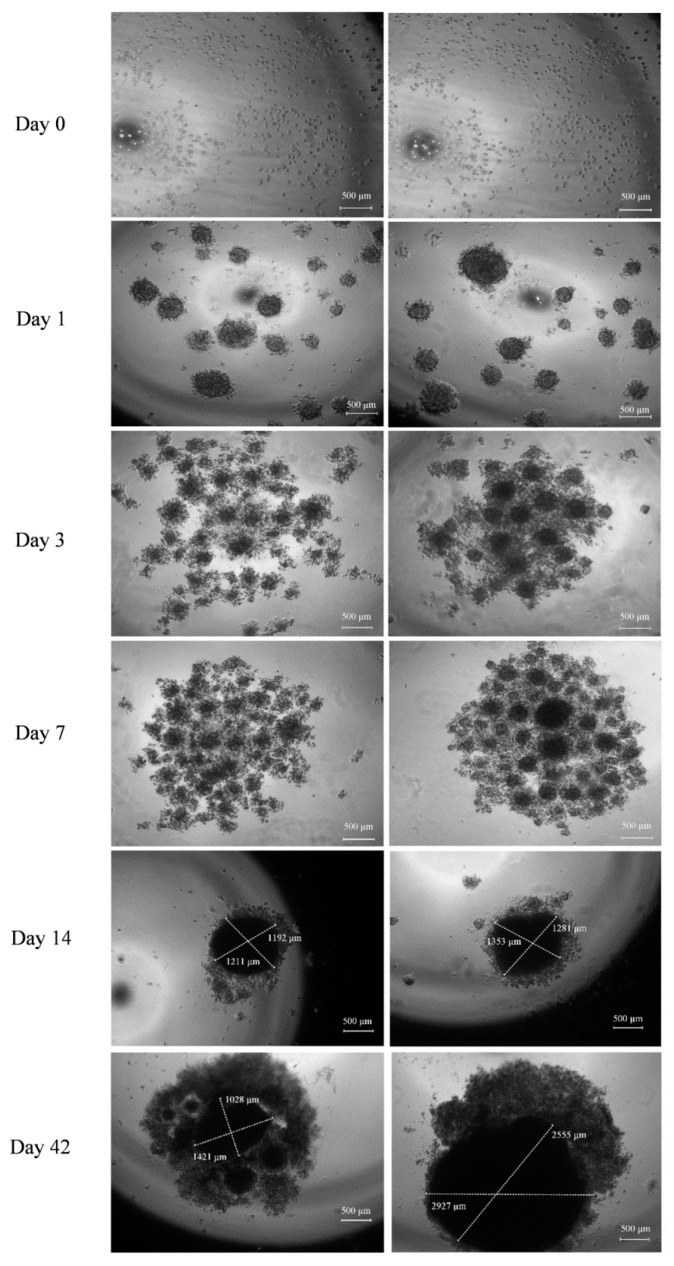
Development of 3D Spheroids Derived from Human Osteoblast Cells. **Left**: Control spheroids without BMP-Phage. **Right**: Spheroids with BMP-Phage. Representative images captured at days 0, 1, 3, 7, 14, and 42. Scale bar: 500 µm.

The generation and development of the control model (cells without BMP-Phage) and the 3D model cultured in the presence of the phage cocktail were monitored using optical microscopy and analyzed with fractal analysis. The images (Figure 2) show that initial cellular aggregations were observed in both conditions on days 1 and 3. By day 7, the presence of BMP-Phage facilitated the formation of more compact and tightly aggregated spheroids compared to the control, indicating the effective role of phage peptides in enhancing cell–cell interactions. Quantitative evaluation (*n* = 30 spheroids per condition) revealed that by day 14, spheroids cultured in the presence of BMP-Phage reached an average diameter of ~1317 ± 46 µm, compared to ~1201 ± 13 µm in the control group. The most pronounced difference was observed at day 42, when treated spheroids attained an average diameter of ~2741 ± 186 µm, significantly larger than control spheroids (~1224 ± 197 µm). This progressive enlargement, accompanied by the formation of more compact and structurally cohesive aggregates, suggests that BMP-Phage incorporation promotes enhanced cellular self-organization and supports the architectural maturation of the 3D model. Furthermore, cell viability was confirmed by comparing the 3D model with cell mono-layers (2D), with or without BMP-Phage. Live/Dead staining further confirmed that the spheroids formed with the BMP-Phage cocktail remained viable, without necrotic or apoptotic areas (Supplementary Figures S1 and S2).

**Figure 3:**
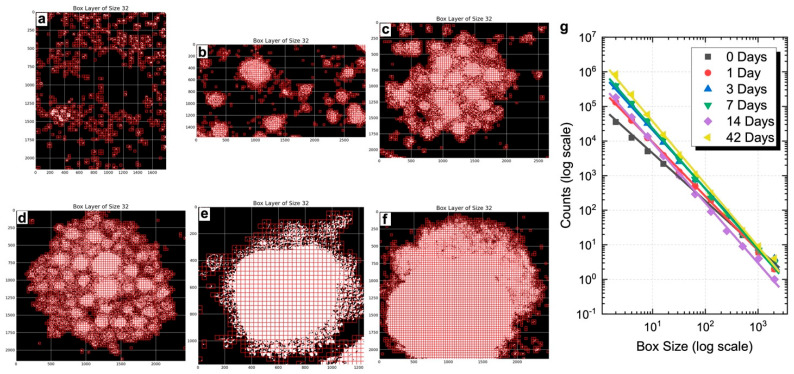
Fractal dimension analysis. Visualization of box layers with size 32: (**a**) Day 0, (**b**) Day 1, (**c**) Day 3, (**d**) Day 7, (**e**) Day 14, (**f**) Day 42 in BMP-Phage spheroids. (**g**) Combined box-counting fractal dimension analysis.

To evaluate whether the 3D spheroids developed morpho-organizational features characteristic of native tissue, a quantitative fractal analysis was performed, and the power law was calculated to assess whether spheroid growth followed a fractal pattern. This method assesses the spatial complexity of biological aggregates by measuring their self-similarity and heterogeneity. FD values were calculated at defined time points (days 0, 1, 3, 7, 14, and 42) using the box-counting algorithm, based on the slope of log–log plots (Figure 3g). FD values are reported as normalized to the morphological baseline of control spheroids processed in parallel. As shown, BMP-Phage spheroids exhibited a progressive increase in FD, from 1.57 at day 1 to 1.81 at day 42, reflecting a time-dependent enhancement in spatial organization and morphological complexity. Representative box-overlay visualizations (Figure 3a–f) illustrate this transition: early-stage spheroids (days 0–3) display fragmented, heterogeneous morphologies, whereas later stages (days 14–42) exhibit compact, cohesive architectures. The most pronounced increase in FD occurred between days 3 and 7, consistent with a phase of active morphogenetic remodeling. From day 14 onward, the FD increment plateaued, indicating a shift toward a more stabilized and organized spheroidal structure. These findings align with microscopy-based qualitative observations and support the role of BMP-Phage in promoting extracellular matrix (ECM) accumulation and 3D architectural maturation.

To complement these findings with a molecular perspective, Raman spectroscopy was employed to assess whether BMP-Phage exposure induced measurable biochemical changes while preserving the structural features of the 3D constructs.

### 2.2. Raman Characterization

**Figure 4:**
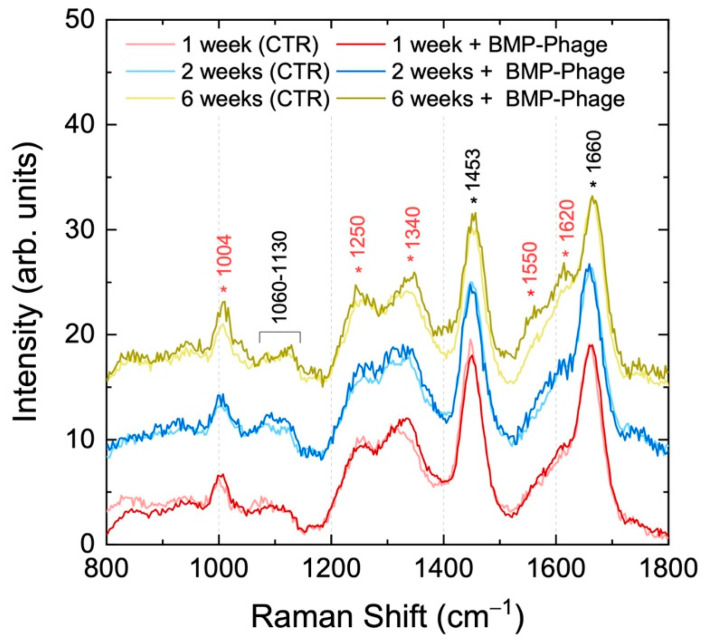
Raman spectra of 3D models with (dark colors) and without (light colors) BMP-Phage up to six weeks.

Raman spectroscopy was employed to assess potential biochemical changes linked to BMP-Phage incorporation and to confirm the preservation of the overall structural integrity of the spheroids, serving as a powerful method for characterizing biological models (Figure 4) [21,22]. Spectra were collected at various time points (from 1 to 6 weeks) within the Raman frequency range of 800–1800 cm^−1^, which encompasses the vibrational fingerprint of biological components. Spectra from samples at different time points are shown in stacking mode, while control spheroids (CTR) and BMP-phage spectra at the same time points are shown superimposed. In all Raman spectra, the observed molecular vibration peaks were predominantly associated with ECM components. Specifically, the peak at 1004 cm^−1^ was attributed to phenylalanine ring breathing mode [23] key structural element of proteins. The vibrational modes between 1060 cm^−1^ and 1130 cm^−1^ were assigned to C–N stretching (ν) [24,25], indicative of glicoprotein components in cell membranes and lipoproteins, characteristic of the ECM. The range from 1240 to 1350 cm^−1^ was associated with a combination of CH_2_ in-plane and out-of-plane bending modes superimposed to the Amide III band, which reflects the protein content of collagen and glycoproteins, crucial for ECM structure [26]. The peak at 1453 cm^−1^ was associated with CH_2_ scissoring (σ) vibration modes in proteins [23,24], while the broad band peaked at 1660 cm^−1^ corresponds to the convolution of Amide I vibrations and in-plane ring stretching modes of aromatic amino acids in proteins [27]. In the Raman spectra of BMP-phage samples, a slight increase in intensity is observed in some bands (highlighted in red in Figure 3) at all time points, as will be commented in the discussion section. Fractal analysis was employed to assess the structural complexity of the 3D spheroids over time. While Raman spectroscopy captured the biochemical fingerprint, fractal analysis, applied to both the 3D structures and their 2D microscopic projections, provided complementary insight into morphological organization and spatial heterogeneity, further enhancing the interpretation of ECM remodelling and tissue-like growth dynamics in response to BMP-Phage treatment.

Table 2 provides a summary of the primary vibrational modes identified in the analyzed samples.

### 2.3. Extracellular Matrix Organization and Matrix Mineralization

**Figure 5:**
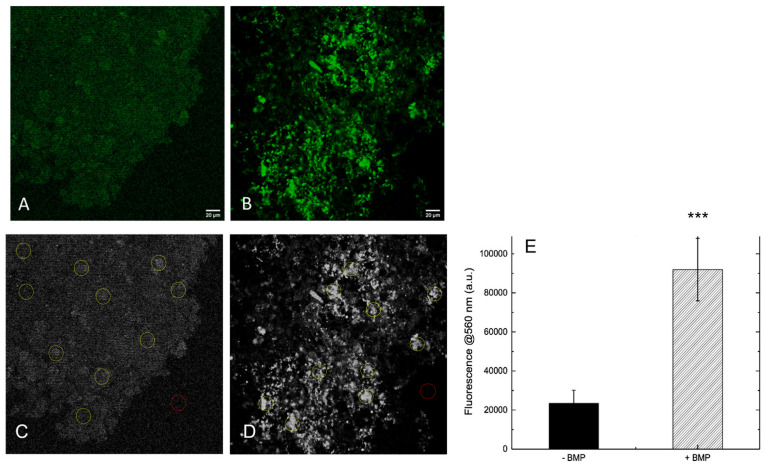
Immunofluorescence staining of SPARC (osteonectin) in 3D models visualized by confocal laser scanning microscopy (green). Representative images of spheroids generated without BMP-Phage (Control), (**A**) and with BMP-Phage (**B**) and quantification of SPARC fluorescence intensity measured at 560 nm (**C**). CLSM images of control spheroids (–BMP) (**C**) and BMP-Phage spheroids (+BMP) (**D**) showing selected regions of interest (ROIs) used for fluorescence quantification (**E**). A dedicated background ROI was included for signal normalization. Bars represent mean ± SD. Statistical analysis was performed using one-way ANOVA followed by Bonferroni post hoc test (*** *p*-values < 0.001). Scale bars: 20 μm.

To evaluate whether the presence of BMP-Phage enhances ECM deposition, both 3D spheroid models were analyzed by immunofluorescent staining of the structural glycoprotein SPARC (osteonectin), a key component involved in ECM remodeling and mineral deposition. Confocal laser scanning microscopy (CLSM) was used to compare ECM accumulation in spheroids cultured with or without BMP-Phage (Figure 5A,B). Confocal images revealed a clear difference in SPARC-specific fluorescence between the two conditions. Control spheroids cultured without BMP-Phage exhibited only low-level, diffuse background fluorescence, possibly attributable to intrinsic signal. In contrast, spheroids cultured with the BMP-Phage cocktail showed markedly higher fluorescence intensity, consistent with enhanced SPARC expression and ECM deposition. This signal increase indicates that BMP-Phage promotes ECM remodelling and contributes to structural maturation in the 3D osteoblast model. In addition, to quantify SPARC-related ECM accumulation, fluorescence intensity at 560 nm was measured across 10 representative regions of interest (ROIs) per sample (Figure 5C,D). A background ROI was included for signal normalization. As shown in Figure 5E, BMP-Phage spheroids displayed a significant increase in fluorescence intensity compared to the control condition, with an average signal approximately four times higher. This quantitative enhancement further confirms the increased ECM deposition and reinforces the role of BMP-Phage in promoting matrix assembly within the 3D osteoblast model.

**Figure 6:**
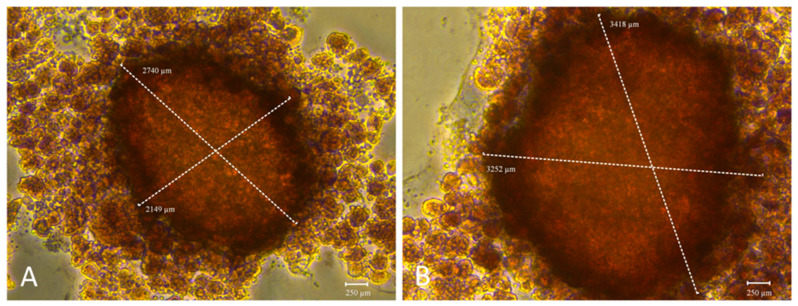
Mineralization evaluation using Alizarin Red S staining. (**A**): 3D spheroids without BMP-Phage. (**B**): Spheroids with BMP-Phage. Scale bar: 250 µm.

**Figure 7:**
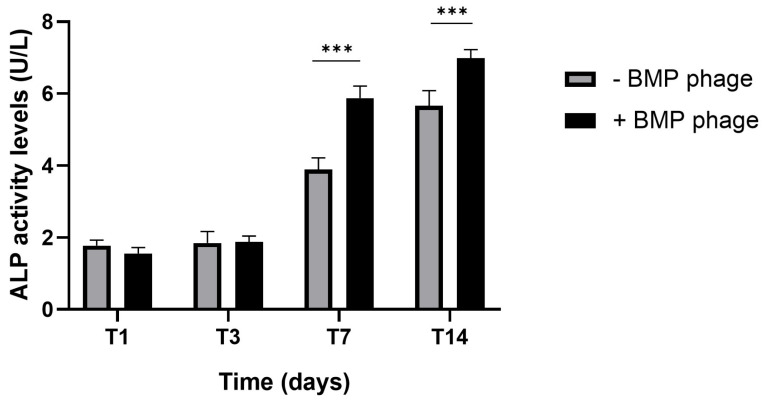
Alkaline phosphatase (ALP) activity in 3D osteoblast spheroids cultured with (+BMP-Phage) or without (–BMP-Phage) BMP-Phage at day 1 (T1), day 3 (T3), day 7 (T7) and day 14 (T14). Data are presented as mean ± SD. Statistical analysis was performed using one-way ANOVA followed by Bonferroni post hoc test (*** *p*-values < 0.001).

The functionality of both 3D models was further assessed by evaluating their mineralization capacity, a key hallmark of osteogenic maturation. To this end, calcium-rich deposits were visualized by staining with Alizarin Red S (ARS) (Figure 6), while alkaline phosphatase (ALP) enzymatic activity was quantified in parallel (Figure 7). These complementary assays were employed to determine whether the addition of BMP-Phage to cells improved the mineralization potential of 3D osteoblastic spheroids. Notably, mineralization analysis revealed increased calcium deposition and a larger mineralization in the BMP-Phage 3D model (3418 µm × 3252 µm) compared to the control (2740 µm × 2149 µm). These findings suggest that the BMP-Phage cocktail effectively promotes extracellular matrix deposition and enhances mineralization, highlighting its potential to support and improve osteogenic differentiation within the 3D model.

To complement the evaluation of mineralized matrix deposition, alkaline phosphatase (ALP) enzymatic activity was quantified from day 1 to day 14 in both BMP-Phage and control spheroids (Figure 7). A marked increase in ALP activity was observed at day 14 in the BMP-Phage, with values reaching (±1.5 fold) compared to the control. 

### 2.4. Gene Expression Profile 

**Figure 8:**
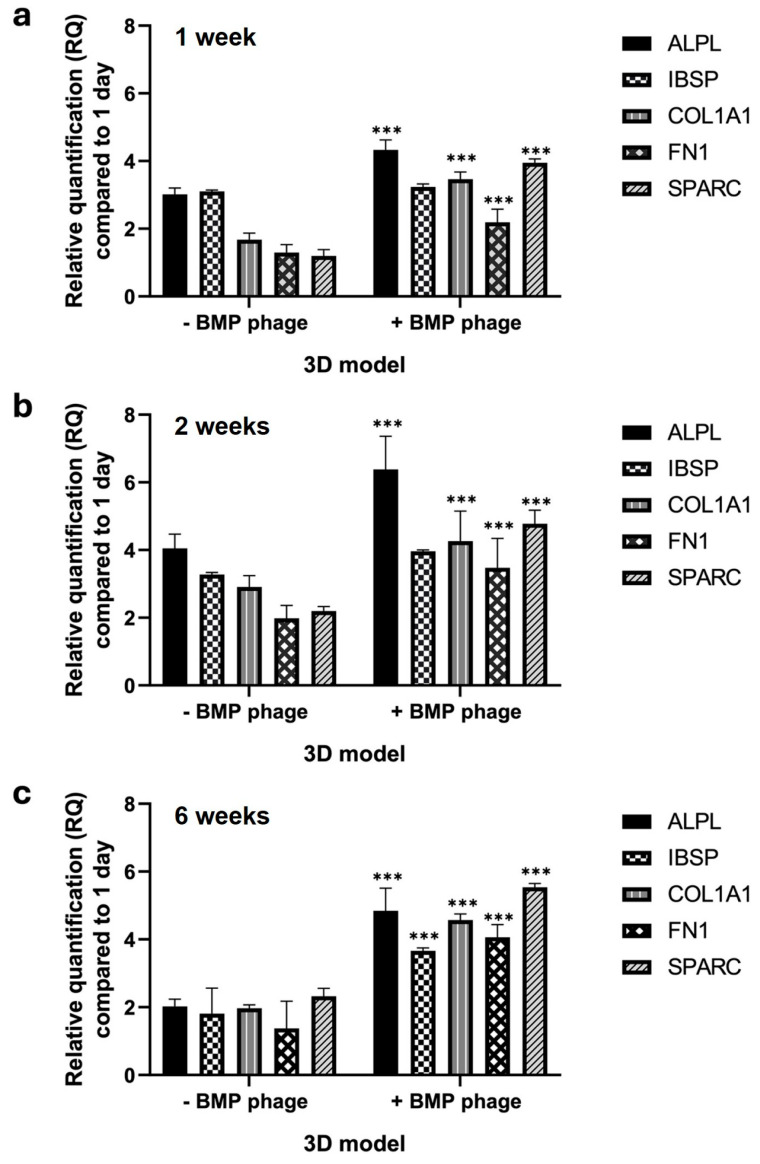
Gene expression analysis of *ALPL*, *IBSP*, *COL1A1*, *FN1*, and *SPARC* in 3D models using qRT-PCR. (**a**) 1 week, (**b**) 2 weeks, and (**c**) 6 weeks in 3D BMP-Phage spheroids (+BMP-Phage) and respective controls (-BMP-Phage). Data were normalized to GAPDH and expressed as fold changes relative to day 1. The statistical analysis was reported as *** *p*-values < 0.001. The data were derived from three independent experiments conducted in triplicate.

The gene expression profile was assessed in both models over a period of six weeks by analyzing key osteogenic markers involved in bone development, differentiation, proliferation, and ECM formation (Figure 8). The markers included Alkaline Phosphatase (*ALPL*), Bone Sialoprotein (*IBSP*), Collagen Alpha-1 Chain Type I (*COL1A1*), Fibronectin 1 (*FN1*), and Osteonectin (*ON*), also known as Secreted Protein Acidic and Rich in Cysteine (*SPARC*) (Figure 6). After one week, spheroids cultured in the presence of the BMP-Phage cocktail exhibited upregulation of *ALPL*, *COL1A1*, *FN1*, and *SPARC*, with an average increase of approximately 2.3-fold compared to the control model without BMP-Phage. This upregulation indicates early stimulation of osteogenic activity triggered by the bone-mimicking peptides. At two weeks, the trend persisted, with a particularly notable increase in *ALPL*, which was upregulated by approximately 2.9-fold relative to the control. This finding suggests sustained activation of pathways involved in osteogenesis and ECM organization. By six weeks, gene expression levels in the 3D control model remained low. However, in the BMP-Phage-treated 3D model, the expression of *IBSP*, *COL1A1*, *FN1*, and *SPARC* showed significant increases compared to the controls. These results highlight the prolonged effect of the BMP-Phage cocktail in enhancing osteogenic pathways and underscore its role in promoting ECM organization and supporting osteogenic differentiation.

## 3. Discussion

This study presents an innovative strategy that employs bacteriophages engineered to display peptides mimicking bone matrix proteins, with the aim of replicating the extracellular matrix (ECM) environment necessary for osteoblast function. This biomolecular approach enabled the development of 3D spheroids of human osteoblasts, offering a physiologically relevant in vitro model capable of supporting osteogenic differentiation, ECM deposition, and mineralisation [30,31].

Spheroids cultured in the presence of BMP-Phage displayed more compact morphology and increased structural cohesion compared to spheroids cultured without BMP-Phage. Morphological analysis showed that by day 42, spheroids in the presence of BMP-Phage reached an average diameter of approximately 2741 ± 186 µm, while those cultured without BMP-Phage measured around 1224 ± 197 µm. This size difference, together with the formation of more cohesive aggregates, suggests that the BMP-Phage cocktail may enhance cellular self-organisation and support the architectural development of the 3D model.

To evaluate the spatial complexity of the spheroids, FD analysis was performed using a box-counting algorithm. Spheroids cultured in the presence of BMP-Phage exhibited a progressive increase in FD values, from 1.57 at day 1 to 1.81 at day 42. The most pronounced change was observed between days 3 and 7, a period indicative of active morphogenetic remodelling. After day 14, the increase in FD plateaued, reflecting the establishment of a more stabilised and structured spheroidal architecture. These findings support the interpretation that BMP-Phage contributes to the organisation and spatial complexity of osteoblast aggregates over time [32].

The biochemical profile of the spheroids was further investigated using Raman spectroscopy. Spectra collected in the 800–1800 cm^−1^ range revealed that spheroids cultured in the presence of BMP-Phage exhibited slightly increased intensity at several key vibrational bands across all time points when compared to those cultured without BMP-Phage. Enhanced signals were particularly evident for the Amide III region (1240–1350 cm^−1^), CH_2_ bending modes (twisting, wagging, and rocking) around 1340 cm^−1^, Amide II (~1550 cm^−1^), and aromatic amino acid ring-stretching vibrations (1570–1620 cm^−1^). At six weeks, the phenylalanine ring breathing mode at 1004 cm^−1^ and CH_2_ scissoring vibration at 1453 cm^−1^ also showed greater intensity. These variations reflect the sustained production and stabilisation of ECM components including proteins, glycoproteins, and lipids [21,22,24], highlighting the impact of BMP-Phage on biochemical remodelling within the 3D environment.

To assess ECM organisation more directly, SPARC (osteonectin) immunofluorescence was examined. Spheroids cultured in the presence of BMP-Phage showed markedly higher SPARC-specific fluorescence compared to those cultured without BMP-Phage. Quantitative analysis revealed an approximately fourfold increase in fluorescence intensity at 560 nm, confirming enhanced ECM accumulation and suggesting a role for BMP-Phage in promoting matrix assembly and maturation [33,34].

The mineralisation potential of the 3D models was also examined. Spheroids cultured in the presence of BMP-Phage exhibited greater calcium deposition, as evidenced by Alizarin Red S staining, with mineralised areas measuring 3418 µm × 3252 µm compared to 2740 µm × 2149 µm in the spheroids cultured without BMP-Phage. These data were corroborated by ALP activity assays, which showed significantly increased enzymatic levels at day 14 in the BMP-Phage condition. Together, these results indicate that the addition of BMP-Phage promotes osteogenic processes such as ECM mineralisation and enzyme activity related to osteoblast differentiation [35,36,37].

Gene expression profiling further supported the functional differentiation of the 3D osteoblast models. After one week, spheroids cultured in the presence of BMP-Phage exhibited upregulation of *ALPL*, *COL1A1*, *FN1*, and *SPARC*, with an average fold change of approximately 2.3 compared to those cultured without BMP-Phage. At two weeks, ALPL expression further increased to approximately 2.9-fold, consistent with the activation of early osteogenic pathways. By six weeks, *IBSP*, *COL1A1*, *FN1*, and *SPARC* remained significantly elevated, indicating sustained osteogenic stimulation and progressive ECM organisation [36,37,38].

An important consideration is the dual function of the phage construct: the filamentous architecture provides structural cues, while the displayed peptides mimic functional domains of native bone matrix proteins [39]. These combined properties may facilitate interactions with cell surface receptors and trigger intracellular signalling cascades. These results are in accordance with previous studies which have shown that phage-based systems can activate osteogenic pathways including BMP/Smad and Wnt/β-catenin, contributing to matrix remodelling and osteoblast differentiation [40,41,42,43,44,45]. This duality underscores the multifunctionality of the BMP-Phage system in promoting both physical organisation and molecular activation within the 3D osteoblast environment.

In summary, the 3D model engineered filamentous phages displaying bone-mimetic peptides demonstrates enhanced spheroid formation, ECM complexity, mineralisation, and osteogenic gene expression over time. The model remains biochemically stable, structurally cohesive, and biologically active for extended culture periods. These characteristics support its application as a reliable and versatile in vitro platform for studying bone tissue physiology and for evaluating biomaterials under controlled, tissue-relevant conditions. 

## 4. Materials and Methods

### 4.1. Cell Culture

The human fetal osteoblast cell line (hFOB 1.19) used in this study was obtained from the American Type Culture Collection (ATCC, Manassas, VA, USA, catalog number CRL-11372; Cellosaurus database accession CVCL_3708). hFOB 1.19 cells were cultured in a 1:1 mixture of Ham’s F12 Medium and Dulbecco’s Modified Eagle Medium (D8437, Sigma, Life Science, Gillingham, UK), supplemented with 2.5 mM L-glutamine (G7513), 0.3 mg/mL G418 (4727878001, Merck Life Science, Milan, Italy), 10% fetal bovine serum (F7524), and 1% penicillin/streptomycin/amphotericin (P4333). Cultures were maintained in a humidified atmosphere containing 5% CO_2_ at 37 °C. The medium was replaced twice a week, and cells were subcultured upon reaching approximately 80% confluence [46,47].

### 4.2. Phage Display Selection, Amplification, and Bioinformatic Analysis

A 9-mer M13 phage display library was constructed in the pC89 vector by cloning random DNA inserts in-frame with the gene encoding the major coat protein pVIII, and subjected to biopanning through incubation with primary human bone marrow-derived cells to isolate phages with high binding affinity, as previously described [19,20]. Biopanning involved multiple rounds of selection with progressively increasing severity, in order to enrich phage clones with high specificity and affinity for the target cells surface. After each round, bound phages were eluted under acidic conditions, amplified in *Escherichia coli* TG1, and titrated. Recombinant clones were identified by blue/white screening on LB agar plates supplemented with X-Gal, IPTG, and ampicillin (Sigma-Aldrich, St. Louis, MO, USA). Blue positive colonies, indicative of phage clones harboring peptide inserts, were isolated after the third round and analyzed individually. DNA inserts from selected clones were amplified by PCR using M13-specific primers and subsequently sequenced. The amino acid sequences were aligned according to their similarity by using the Clustal X 2.1 (available at http://clustalx.software.informer.com/2.1/, accessed on 15 April 2024) GeneDoc (available at https://genedoc.software.informer.com/amp/2.7/, accessed on 15 April 2024) was used as a tool for visualizing, editing and analyzing multiple sequence alignments of the peptides. Alignment similarity for each peptide were determined through BLASTp (BLAST suite, https://blast.ncbi.nlm.nih.gov/Blast.cgi, accessed on 15 April 2024) analysis against protein databases (taxid: 9606, Homo sapiens), and the corresponding literature references have been reported in Table 1.

### 4.3. ELISA Assay for Phage Clone Characterization

A cell-based ELISA was performed to assess the binding specificity of phage-displayed peptides to human osteoblast cells. hFOB 1.19 cells were seeded in 96-well plates at a density of 5 × 10^5^ cells/well in phosphate-buffered saline (PBS; Sigma-Aldrich, St. Louis, MO, USA) and incubated overnight at 4 °C to allow cell attachment. Cells were then fixed with 4% paraformaldehyde (PFA) (Merck Life Science, Milan, Italy) for 10 min at room temperature. After fixation, plates were washed three times with PBS containing 0.05% Tween-20 (PBS-T).

Non-specific binding was blocked by incubating cells with 300 μL of PBS supplemented with 5% non-fat dry milk and 0.05% Tween-20 for 2 h at 37 °C under agitation. Following a brief wash with PBS-T, 100 μL of purified phage clones (1 × 10^11^ TU/mL), diluted in PBS with 1% milk and 0.1% Tween-20, were added to each well and incubated for 1 h at 37 °C with agitation.

After five washes with PBS-T, bound phages were detected by adding 100 μL/well of anti-M13 HRP-conjugated antibody (1:5000 dilution in PBS containing 1% milk and 0.1% Tween-20), followed by incubation for 1 h at 37 °C under agitation. Plates were then washed five times and developed with 100 μL/well of TMB (Sigma-Aldrich, St. Louis, MO, USA) substrate. The enzymatic reaction was stopped with 1 M HCl, and absorbance was measured at 450 nm using a microplate reader (Multiskan GO, Thermo Scientific, Waltham, MA, USA). Mouse fibroblast cells (L929) were used as negative controls to assess non-specific interactions. Additionally, a phage clone displaying a non-specific peptide sequence (K–) was included as a functional negative control to validate the specificity of the selected BMP-Phage clones. This K– phage-displayed peptide confirmed that the observed effects were specifically attributable to the selected BMP-related peptides.

### 4.4. 3D Human Osteoblast Model Generation and Microscopic Characterization

#### 4.4.1. Preparation of Hanging Drops

The 3D spheroid models were generated using the hanging drop method [48,49]. Control samples were prepared at a concentration of 2.5 × 10^6^ cells/mL without the addition of BMP-Phage, grown under the same conditions as treated ones, with equivalent volumes of medium and phosphate-buffer saline (PBS) used for phage resuspension. Experimental samples were prepared by suspending cells in growth medium supplemented with BMP phage at a final concentration of 10^11^ TU/mL. Drops were monitored at specific time points (1, 3, 7, 14, and 42 days). Each condition was tested in triplicate and repeated in three independent experiments to ensure reproducibility.

#### 4.4.2. Microscopic Analysis

The hanging drops were monitored daily throughout the incubation period to observe the formation of cell sheets or aggregates. Representative images of each aggregation stage (magnification: 40×) were captured at multiple time points (0–42 days) using a Leica DMi1 inverted microscope equipped with a FLEXACAM C1 12 MP stand-alone camera (Leica Camera AG, Wetzlar, Germany). Images were processed using Capture 2.4 software (New York Microscope Company, Hicksville, NY, USA).

#### 4.4.3. Fractal Dimension (FD) Analysis

Fractal dimension was calculated using the FracLac plugin for ImageJ (Version 1.54d; National Institutes of Health, Bethesda, MD, USA; available at https://imagej.nih.gov/ij/plugins/fraclac/; accessed on 23 June 2024) by applying a box-counting algorithm to binarized images. All images were processed under identical conditions, and BMP-Phage spheroid measurements were normalized to the corresponding control spheroids analyzed in parallel.

FD was calculated using the Box Counting method, which involves overlaying grids of progressively smaller boxes on spheroid images and counting the number of boxes that intersect the structure at each scale. For this purpose, a standard measurement unit was applied.

To prepare the images, spheroid samples were pre-processed to reduce noise and isolate objects from the background by thresholding, converting them into binary format. The total segment length (L) was determined using the following Equation (1):

*L* = *Nε* (1)

where N is the number of units of size *ε* needed to cover the segment. 

Surface and volume were calculated similarly, leading to the general Equation (2):

*L*_2_ = *Nε*_2_ (2)

In a similar way, it can be calculated the volume (3):

*L*_3_ = *Nε*_3_ (3)

and in general

*L*_D_ = *Nε*_D_ (4)

where D represents the object’s topological dimension. 

Clearing D in (4), the following equation can be obtained (5):

(5) $$D=\frac{ln(N)}{ln\left(\frac{L}{\varepsilon }\right)}$$

which can also be written as follows and calls $D_{f}$:

(6) $$D_{f}=\frac{ln(N)}{ln\left(\frac{L}{\varepsilon }\right)}$$

Fractal analysis [50] is aimed at characterizing objects through the calculation of the FD, which is particularly useful for analysing medical images (MI). One of the most used methods for calculating FD, due to its simplicity and efficiency when sufficient images are available, is the Box Counting (BC) method.

In the BC method, an image of dimensions *M* × *M* (M = 2k) is divided into a grid of windows of decreasing sizes:

(7) $$R=\frac{M}{\varepsilon },\left(R=2^{k},i=0..k\right)$$

**Figure 9:**
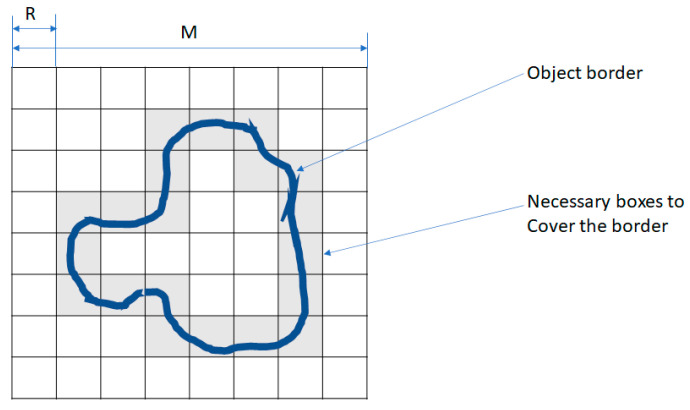
Box Counting (BC) method. Example showing 20 boxes required to cover the entire object boundary.

The number of windows required to cover the object, N(R), is counted for each scale, as shown in Figure 9. The FD is then calculated using the following equation:

(8) $$D_{f}=\frac{ln(N\left(R\right))}{ln(R)}$$

where R≠1

#### 4.4.4. Raman Characterization

Samples were transferred onto CaF_2_ slides to minimize fluorescence interference from the substrate. They were washed with PBS, fixed with 4% formalin for 20 min, and rinsed with ultrapure water before Raman spectroscopy analysis.

Raman spectra were acquired using a 473 nm laser (COBOLT) focused at 0.5 mW through a 100× objective (N.A. 0.9) mounted on an Olympus microscope. Backscattered signals were collected over 10 s using a Horiba iHR550 spectrometer equipped with a 600 lines/mm diffraction grating and a CCD detector (Syncerity, Horiba, Kyoto, Japan). Data analysis was performed using Horiba LabSpec software (Labspec 5).

### 4.5. Extracellular Matrix Organization and Mineralization 

#### 4.5.1. Immunofluorescence Analysis 

CLSM was used to analyze ECM organization in the 3D spheroid samples after one week of incubation. SPARC (osteonectin) polyclonal antibodies (Invitrogen) were applied to label the ECM, followed by secondary anti-rabbit IgG AlexaFluor 555-conjugated antibodies (Invitrogen). Samples were mounted on microscope slides and observed using a Leica DMIRE2 inverted microscope coupled with a TCS SP2 confocal system. Fluorescence was excited at 514 nm and collected at 560 nm to avoid laser reflection. Images were acquired at 63× magnification [51] and processed using ImageJ software (available at https://imagej.net/ij/, accessed on 27 June 2024). (NIH, Bethesda, MD, USA). To quantify ECM-associated fluorescence in CLSM images, each image was first converted to RGB format and the green channel was extracted. Ten regions of interest (ROIs) were selected per image to measure mean fluorescence intensity. Background signal was measured from a dedicated ROI and used for normalization. The normalized fluorescence values were averaged across samples and the resulting data were plotted (see Figure 5).

#### 4.5.2. Alizarin Red S Assay

To evaluate matrix mineralization, calcium deposits were stained with Alizarin Red S (AR-S). After 14 days of incubation, samples were washed twice with PBS (Ca^2+^/Mg^2+^-free) and fixed with 4% formaldehyde for 15 min. The cells were stained with 1% AR-S solution (pH 4.2) for 20 min, followed by rinsing three times with deionized water. Images were captured using a Leica inverted microscope equipped with a FLEXACAM C1 12 MP camera [52].

#### 4.5.3. Alkaline Phosphatase Activity Assay

Alkaline phosphatase (ALP) enzymatic level was measured using a colorimetric method based on the conversion of p-nitrophenyl phosphate into p-nitrophenol using Alkaline Phosphatase Assay Kit (ABCAM ab83369). The assay was acquired at 405 nm using a microtiter plate reader (Multiskan GO, Thermo Scientific, Waltham, MA, USA) and expressed as ALP level (nmol) obtained by standard curve according to the manufacturer’s instructions. 

#### 4.5.4. Gene Expression Analysis

The expression of key osteogenic markers (ALPL, IBSP, COL1A1, FN1, and SPARC) was analyzed during spheroid development at 7, 14, and 42 days in both control and BMP-Phage-treated models. Total RNA was extracted using TRIzol reagent (Invitrogen, Carlsbad, CA, USA) and quantified using a NanoDrop ND-1000 UV spectrophotometer. qRT-PCR was performed using SsoAdvanced Universal SYBR Green Supermix (Bio-Rad Laboratories, Hercules, CA, USA) in a 20 μL reaction containing 1 μL of cDNA, 0.5 μM of forward and reverse primers, and 10 μL of supermix.

Amplification was performed on a 7500 Fast Real-Time PCR System, and melting curve analysis was conducted using default settings. Gene expression data were analyzed using the 2^−ΔΔCt^ method, normalized to GAPDH, and expressed as fold changes relative to day 1. Oligonucleotide sequences are listed in Table 3 [52].

### 4.6. Statistical Analysis

All experiments were conducted in triplicate and repeated at least three times. Results are expressed as mean ± standard deviation. Data were analyzed using GraphPad Prism version 8.0.1 (GraphPad Software, San Diego, CA, USA). Statistical significance was determined using one-way ANOVA followed by Bonferroni post hoc tests for multiple comparisons. *p* ≤ 0.05 was considered statistically significant. 

## 5. Conclusions

The 3D osteoblast model developed in this study, based on a mixture of filamentous bacteriophages displaying bone-mimic peptides, successfully supported osteogenesis, ECM synthesis, and mineral deposition. The system demonstrated sustained structural integrity, biochemical stability, and long-term cellular functionality. These characteristics establish it as a robust and versatile in vitro platform for investigating bone tissue physiology in controlled microenvironments.

This model also offers a valuable foundation for advancing research in regenerative medicine. Future developments should focus on its integration into dynamic culture platforms, such as microfluidic organ-on-a-chip systems, to enhance physiological relevance in future translational studies and enable applications in translational and precision medicine.

## Figures and Tables

**Table 1:** Sequence similarity of phage-displayed peptides with bone matrix proteins.

Peptide Sequence	Assignment	References
GRKAANASS	Bone Morphogenetic Protein 2 (BMP-2)	[19]
QRRAGPVPP	Osteonectin (SPARC)	[15]
NRKGTSTNL	Integrin-Binding Sialoprotein (IBSP)	[19]
MKKAWPGRA	Collagen alpha-1(I) chain	[19]
SRRIGPTAP	Fibronectin	In this work

**Table 2:** Raman peak assignments for the analysed organoids.

Frequency (cm^−1^)	Vibrational Mode	Assignment	Reference
1004	ν C=C Ring breathing mode	Phenylalanine	[24]
950	ν C–C	Proteins, Lipids	[24]
1060–1130	ν C–N	Proteins	[24,25]
1240–1350	τ CH_2_ (twisting), Amide III (α-helix, β-sheet)	Proteins, Lipids	[26,28,29]
1455	σ CH_2_ (scissoring)	Proteins, Lipids	[23,24]
1550–1620	Amide II ν C=C Ring Amide I (antiparallel β-sheet)	Proteins Phenylalanine, Tyrosine, Tryptophan	[23,27,29]
1660	Amide I (α-helix)	Proteins	[26,28]

**Table 3:** Primer sequences (5′–3′) used for qRT-PCR analysis.

Protein Name	Target Gene	Protein Function	Forward	Reverse
Glyceraldehyde 3-phosphate dehydrogenase	*GAPDH*	Housekeeping genes	AACAGCGACACCCACTCCTC	CATACCAGGAAATGAGCTTGACAA
Alkalinephosphatase	*ALPL*	Bone mineralization	ACCATTCCCACGTCTTCACATTT	AGACATTCTCTCGTTCACCGCC
Bone Sialoprotein	*IBSP*	Cell adhesion	GGCAGTAGTGACTCATCCGAAG	GAAAGTGTGGTATTCTCAGCCTC
Osteonectin	*ON*	Binding of calcium-ECM and collagen assembly	TGCCTGATGAGACAGAGGTGGT	CTTCGGTTTCCTCTGCACCATC
Collagen Alpha 1 Chain Type I,	*COL1A1*	Bone tissue integrity e structure	CCCTGCCAGATCTGTGTCTG	GTGGTTTCCTGGTCGGTGG
Fibronectin,	*FN1*	Migration and matrix assembly	ACAACACCGAGGTGACTGAGAC	GGACACAACGATGCTTCCTGAG

## Data Availability

All data supporting the findings of this study are available within the document.

## Supplementary Materials

The following supporting information can be downloaded at: https://www.mdpi.com/article/10.3390/ijms26178482/s1, Figure S1: Cell viability of hFOB 1.19 cells cultured in 2D or 3D, with or without BMP-Phage cocktail, assessed by MTT assay. Figure S2: Representative image of a spheroid cultured with BMP-Phage cocktail for 6 weeks, stained with Live/Dead assay and DAPI. Supplementary Methods: MTT assay for cell viability; Live/Dead staining merged with DAPI.
