# Supplementary material for: Formation of 3D Human Osteoblast Spheroids Incorporating Extracellular Matrix-Mimetic Phage Peptides as a Surrogate Bone Tissue Model
Source: Int J Mol Sci. 2025 Sep 1;26(17):8482. doi: 10.3390/ijms26178482 (PMC12428906; doi:10.3390/ijms26178482)
Supplement: Supplementary file 1 [file ijms-26-08482-s001.zip › ijms-3790511-supplementary.pdf]

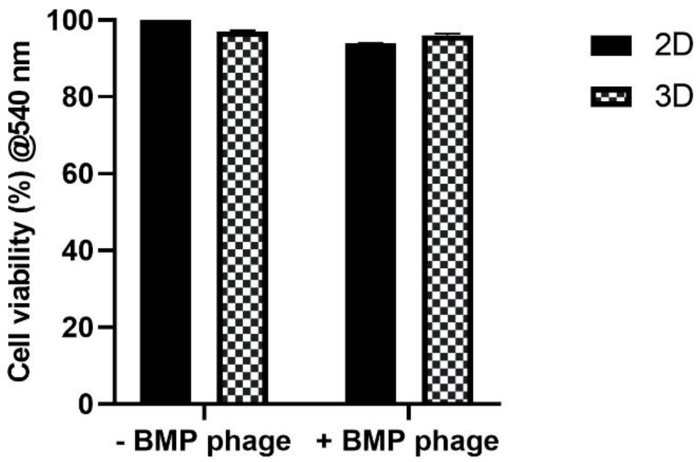

Supplementary Figure S1. Cell viability of hFOB 1.19 cells cultured in 2D and 3D, with and without BMP-Phage cocktail, assessed by MTT assay. Data are expressed as mean ± SD from $n = 3$ independent experiments.

No significant differences in cell viability were detected between 2D and 3D model, regardless of BMP-Phage treatment, confirming that the system is well tolerated under both culture conditions.

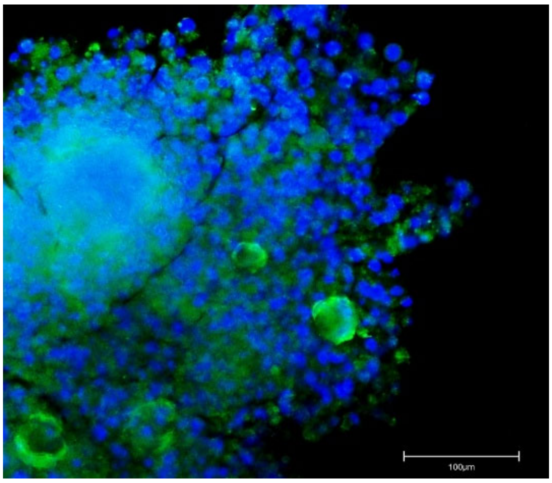

Supplementary Figure S2. Representative image of a spheroid cultured with BMP-Phage cocktail for 6 weeks, stained with Live/Dead assay and DAPI

Live/Dead staining demonstrated that 3D spheroids cultured with the BMP-Phage cocktail remained fully viable, without necrotic or apoptotic zones, throughout the experimental period, including the final time point.

Supplementary Methods

MTT assay for cell viability

Cell viability was assessed using the MTT [3-(4,5-dimethylthiazol-2-yl)-2,5-diphenyltetrazolium bromide] (Sigma-Aldrich, M2128, St. Louis, MO, USA) assay as an indirect indicator of cell metabolic activity. hFOB 1.19 cells were cultured under 2D (standard cells culture plates) and 3D (hanging drop method) conditions, with or without BMP-Phage cocktail. After 24 hours, cells were incubated with MTT solution (1 mg/mL) for 2 hours at 37 °C. Formazan crystals were solubilized in DMSO (Sigma-Aldrich, St. Louis, MO, USA), and absorbance was measured at 540 nm using a microplate reader (BioTek Instruments, Inc., VT, USA)). Results are expressed as percentage of viable cells relative to controls (2D). All experiments were performed in triplicate.

Live/Dead staining merged with DAPI

Cell viability within samples was assessed using a Live/Dead Viability Kit (Thermo Fisher Scientific, USA), based on staining with SYTO 9 and propidium iodide (PI). The cells were incubated with 5 µM SYTO 9 and 30 µM PI in PBS for 30 minutes at 37°C in the dark. The nuclei were stained with Mounting Medium with DAPI—Aqueous Fluoroshield (AB104139, Abcam, Milan, Italy), and observed using a Leica DMi1 inverted microscope equipped with a FLEXACAM C1 12 MP stand-alone camera (Leica Camera AG, Wetzlar, Germany).
